# Single Superficial versus Dual Systems Venous Anastomoses in Radial Forearm Free Flap: A Meta-Analysis

**DOI:** 10.1371/journal.pone.0134805

**Published:** 2015-08-13

**Authors:** Shuang Bai, Zhong-Fei Xu, Wei-Yi Duan, Fa-Yu Liu, Dong-Hui Huang, Chang-Fu Sun

**Affiliations:** 1 Department of Oromaxillofacial-Head and Neck Surgery, School of Stomatology, China Medical University, No.117, Nanjing North Street, Heping District, Shenyang, Liaoning, People’s Republic of China; 2 Department of Oral and Maxillofacial Surgery, School of Stomatology, China Medical University, No.117, Nanjing North Street, Heping District, Shenyang, Liaoning, People’s Republic of China; 3 Department of Clinical Epidemiology, the first affiliated hospital of China Medical University, No.155, Nanjing Street, Heping District, Shenyang, Liaoning, People’s Republic of China; Bascom Palmer Eye Institute, University of Miami School of Medicine;, UNITED STATES

## Abstract

**Background:**

The radial forearm free flap (RFFF) has been widely used with increasing frequency in head and neck reconstruction following extirpative surgery. The controversy of the venous anastomoses patterns still exists. Thus, we conducted a meta-analysis to assess the relationship between the venous anastomoses patterns and venous compromise.

**Methods:**

MEDLINE, PubMed, Web of Science, and Wanfang databases were searched for studies reporting the different venous anastomoses patterns of the RFFF. A meta-analysis was conducted using the random effects models. Publication bias and sensitivity analysis were also assessed.

**Results:**

6 studies with 992 cases were included in this meta-analysis. The dual anastomosis group tended to have a lower incidence of venous compromise (RR = 1.39). However, the difference was not statistically significant (95%CI: 0.59, 3.24).

**Conclusions:**

This meta-analysis indicated that performing dual venous anatomoses consisting of superficial and deep systems conferred a tendency of the reduction with regard to venous compromise.

## Introduction

Since its original description [1, 2], the radial forearm free flap (RFFF) remains a frequent choice for reconstruction in head and neck surgery. Owing to its thinness, pliability, versatility and a long vascular pedicle with large caliber, easy harvest approach as well the feasibility of two teams performing, RFFF has particularly been suitable for intra-oral and pharyngeal reconstruction.

The RFFF has 2 systems of venous drainage: a deep system composed of 2 venae comitantes accompanying the radial artery and a superficial system composed of the cephalic vein and a series of subcutaneous veins. Although widely studied, the controversy of one versus two venous anastomoses still exists.

Supporters of the superficial system argue that single venous anastomosis of the cephalic vein can shorten operative time, and that does not interfere with the success of the tissue transfer [3]. Some take the opposite position, arguing that the deep system is as capable as the superficial vein in draining the radial forearm flap hemodynamically [4]. Others reported a fail-safe drainage method, which functions in a self-sustaining manner, utilizing two separate flap venous systems and two neck recipient venous systems [5].

The selection of the drainage system largely depends upon the surgeon’s preference and individual flap vasculature. Although the published success rates of RFFF approach 95% [6], a series of complicating factors still can lead to unpredictable venous compromise postoperatively. The debate as to whether the selection of venous drainage is related to the venous crisis is ongoing.

The aim of this paper is to carry out a meta-analysis to assess the incidence of venous compromise of different venous anastomosis patterns.

## Materials and Methods

### Search strategy

MEDLINE, PubMed, Web of Science, Wanfang databases were searched for articles till February 1, 2015 with broad key terms, such as “radial forearm flap,” “venous anastomosis,” “venous anastomoses,” and “venous drainage.” Only studies published in English and Chinese were included. For example, a review of literature was performed targeting the Web of Science database. The search strategy was carried out using the retrieval type as following: “radial forearm flap” AND (“venous anastomosis” OR “venous anastomoses” OR “venous drainage”). It was limited to articles published in English till February 1, 2015, which yielded 133 articles. Manual search of reference lists of retrieved articles was also performed.

### Study selection and inclusion/exclusion criteria

2 reviewers independently performed the study selection. The studies identified through database searching were screened by removing duplications using the Endnote X7 software. The irrelevant studies were excluded by reading titles and abstracts. The selected articles were further assessed for full-text reading. Finally, the eligible articles were included only if they met the following criterions:

1. Studies must contain data of the comparison between the superficial system and the superficial plus deep system. The sole reports about the reliability of the single venous drainage would not be considered.
2. Studies must regard the incidence of venous compromise as the main summary measure.
3. Studies from different periods or departments were included as separate studies.

The exclusion criterions were as following: (1) being a review, comment, or editorial; (2) animal model studies or fresh cadaver dissection studies; (3) duplicated studies; (4) sample size less than 30.

### Literature quality assessment

The quality of all included studies was assessed according to the Newcastle-Ottawa quality Assessment Scale [7] independently by 2 reviewers (Shuang Bai, Zhong-Fei Xu). Disagreements were resolved by another reviewer (Chang-Fu Sun). The Newcastle-Ottawa Quality Assessment Scale for cohort study falls into three categories, including Selection, Comparability and Outcome. The category Selection and Outcome respectively has four and three items. The category Comparability only has one item. When a study is assessed item by item, it is awarded by a maximum of one star (★) for each item within the Selection and Outcome categories. A maximum of two stars can be given for Comparability category. Generally, the study which is awarded more than five stars in total will be considered to be included in this meta-analysis.

### Statistical analysis

The strength of association between venous anastomosis and the venous compromise was estimated by risk ratio (RR) value and 95% confidence interval (CI). A meta-analysis of incidence of venous compromise between the two groups, superficial system and superficial plus deep system, were combined using STATA 11 (Stata, College Station, TX, USA) to obtain a risk ratio. Z-test determined the significance of the pooled RR and P<0.05 was considered as statistically significant. Heterogeneity of the studies was assessed using the Cochran Q and I^2^ statistic [8], which represents the percentage of total variation among studies that attributes to heterogeneity rather than chance [9]. If the study met the hypothesis of homogeneity, the Mantel-Haenszel fixed effects models were used; otherwise, random effects models were utilized to estimate risk ratios for outcomes.

Begg and Egger rank correlation tests were used to assess the extent of publication bias. In addition, sensitivity analysis was also performed. Those two procedures were conducted using STATA 11 (Stata, College Station, TX, USA).

### Ethics Statement

All clinical investigations were conducted according to the principles expressed in the Declaration of Helsinki. The patient who involved in the case presentation provided written informed consent for participation in this research. The individual in this manuscript has given written informed consent (as outlined in PLOS consent form) to publish these case details. The Ethical Committee of China Medical University has specially approved this study.

## Results

### Studies and population

**Fig 1:**
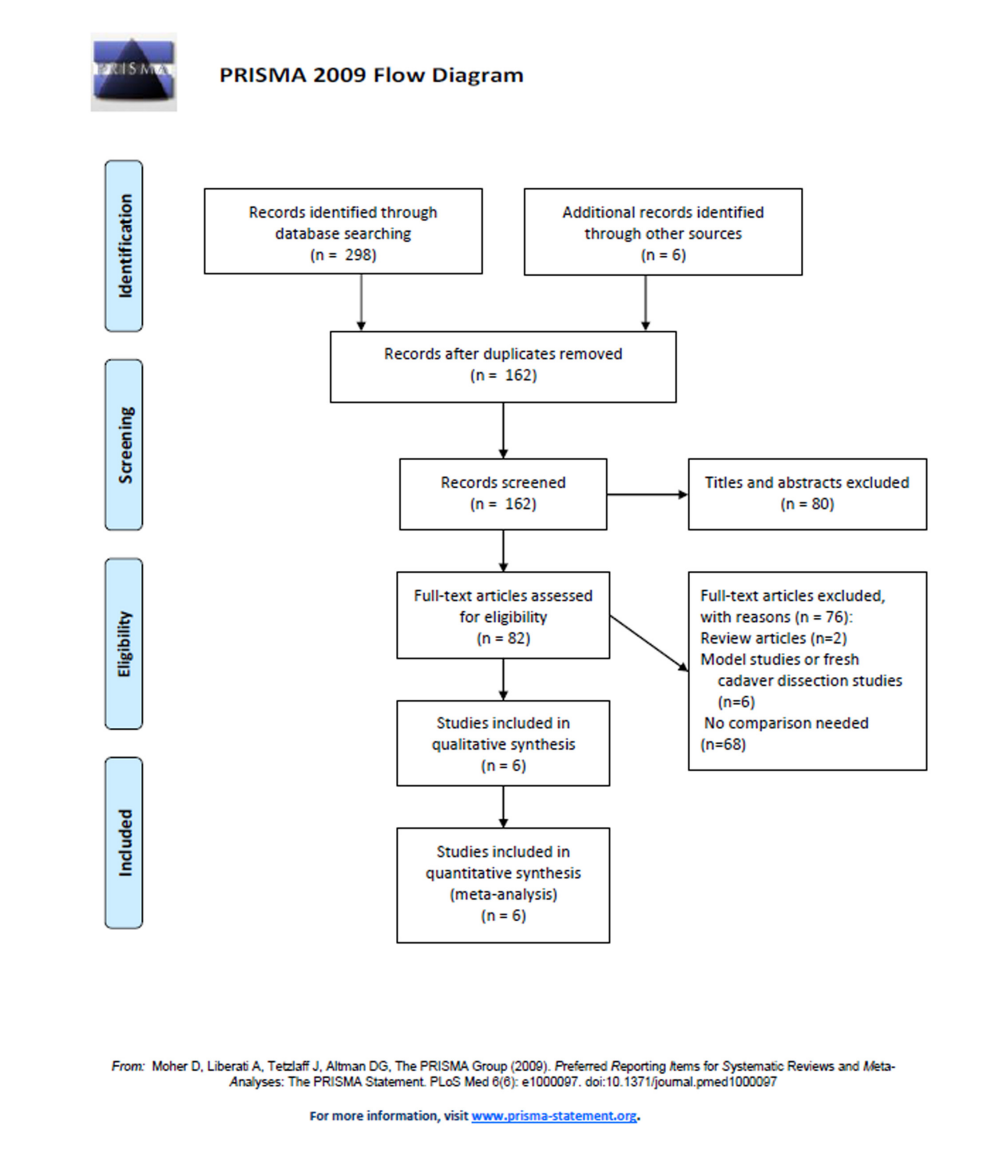
PRISMA flow diagram for the study selection process.

There were some 304 studies identified from the search strategy. These were imported into a bibliographic database by using the Endnote X7 software. The titles and abstracts were screened and 80 studies were excluded at this step. Then, full-text articles were screened against the inclusion criteria. Thus, 6 studies [3, 5, 10–13] with 992 participants were included in our study. We followed the PRISMA guidelines and illustrated the study selection by the PRISMA flow diagram (Fig 1).

The characteristics of included studies are presented in Table 1. 5 of the studies were published in English, and 1 in Chinese. These studies were undertaken all over the world with 1 in Japan, 3 in China, 1 in United States, 1 in Germany. A total of 992 patients were recruited over periods ranging between 1987 to 2011 years. At all clinical centers involving in this meta-analysis, the RFFF was one of the most widely used free flaps in oral and maxillofacial reconstruction. The RFFF was characterized by thinness and pliability and had frequently been selected for intra-oral and pharyngeal reconstruction in the cases involved in this meta-analysis. In their practice, the question of how to select the most effective venous outflow of RFFF aroused their attention, thus, the authors of these studies performed a retrospective cohort study of their databases. All the patients included were operated for malignant tumor resection and reconstruction with the RFFF. All the RFFF were elevated as classic fashion and the conventional postoperative managements were carried out in all cases. Each clinical center instructed experienced surgeons to fulfill the operative procedures, in most cases, the same surgeon carried out the anastomoses in each case series. Thus, we think the studies are comparable according surgical setting and surgeons. Microvascular anastomoses were performed with handmade interrupted sutures in all included cases. The donor site complications included partial skin-graft loss, numbness and dissatisfaction with appearance. The recipient site complications included hematoma, flap loss, infection and fistula, with special attention to venous compromise. The incidence of venous insufficiency or compromise was the outcome used in all the included studies. In all included studies, the conventional postoperative managements were carried out. This is comparable and convention of postoperative monitoring. The flap was monitored every 2 hours for at least 3 days, daily for 2 weeks through clinical assessment of the flap skin color, texture, warmth, capillary refill and pin-prick testing. Emergency exploration was carried out on clinical suspicion of vascular crisis.

**Table 1:** The characteristics of the studies included in this meta-analysis.

First author	Year of publication	Country	No. of single anastomose(superficial)	No. of dual anastomoses(superficial+deep)	Reexploration for venous crisis^a^	Reexploration for venous crisis^b^
Ichinose A	2003	Japan	144	163	15	1
Liu Y	2008	China	68	68	5	6
Selber JC	2011	US	131	37	9	2
Rohleder NH	2011	Germany	9	38	1	1
Liu YF	2012	China	98	80	7	6
Shi RH	2012	China	71	85	1	8

^a^ Data from the group of single superficial anastomose

^b^ Data from the group of dual anastomoses (superficial+deep)

All the included studies showed more than five stars by quality assessment (Table 2). These included studies all illustrated explicit diagnostic criteria, good comparability between subgroups and clear results.

**Table 2:** Result of literature quality assessment according to the Newcastle-Ottawa quality Assessment Scale.

Study ID	Selection	Comparability	Outcome
Ichinose A 2003	★★★★	★★	★★
Liu Y 2008	★★★	★★	★★
Selber JC 2011	★★★★	★★	★★★
Rohleder NH 2011	★★	★★	★★
Liu YF 2012	★★★	★★	★★
Shi RH 2012	★★★	★★	★

### Meta-analysis and pooled incidence of venous compromise

**Fig 2:**
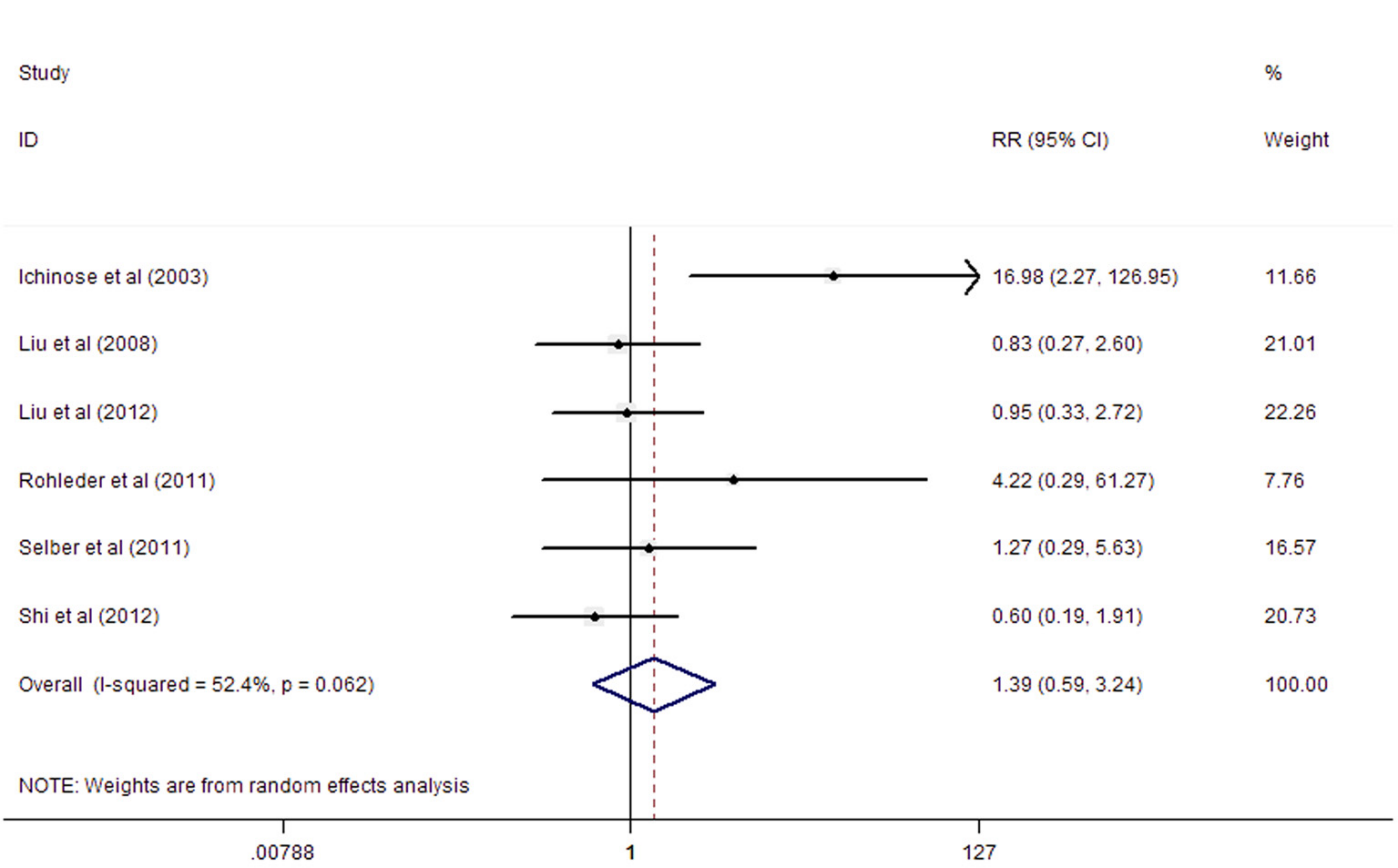
Forest plot for venous compromise. The difference was not statistically significant (RR = 1.39, 95%CI: 0.59, 3.24).

There was substantial heterogeneity with an I^2^ value of 52.4%; thus, random effects models were used. In total, the dual anastomosis group tended to have a lower incidence of venous compromise (RR = 1.39) (Fig 2). However, the difference was not statistically significant (95%CI: 0.59, 3.24). This result indicated that dual anastomosis consisting of the superficial and deep veins showed the tendency to decrease risk of venous compromise.

### Publication bias and sensitivity analysis

**Fig 3:**
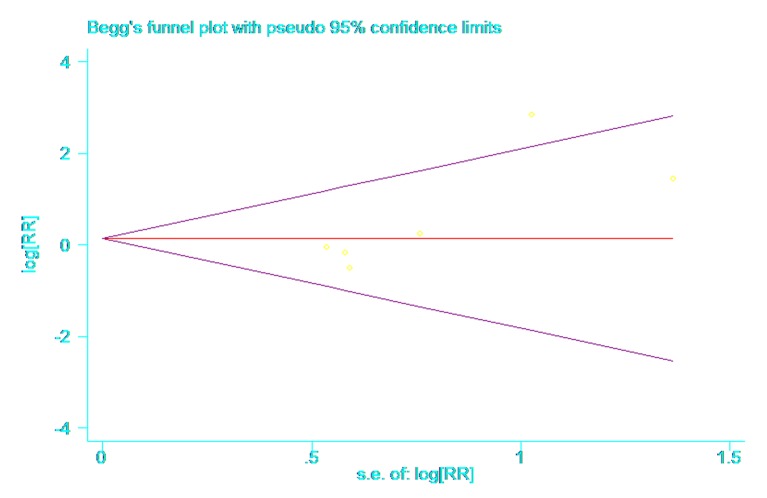
Begg’s funnel plot for publication bias. The result showed that there was no publication bias in this meta-analysis (P = 0.091).

**Fig 4:**
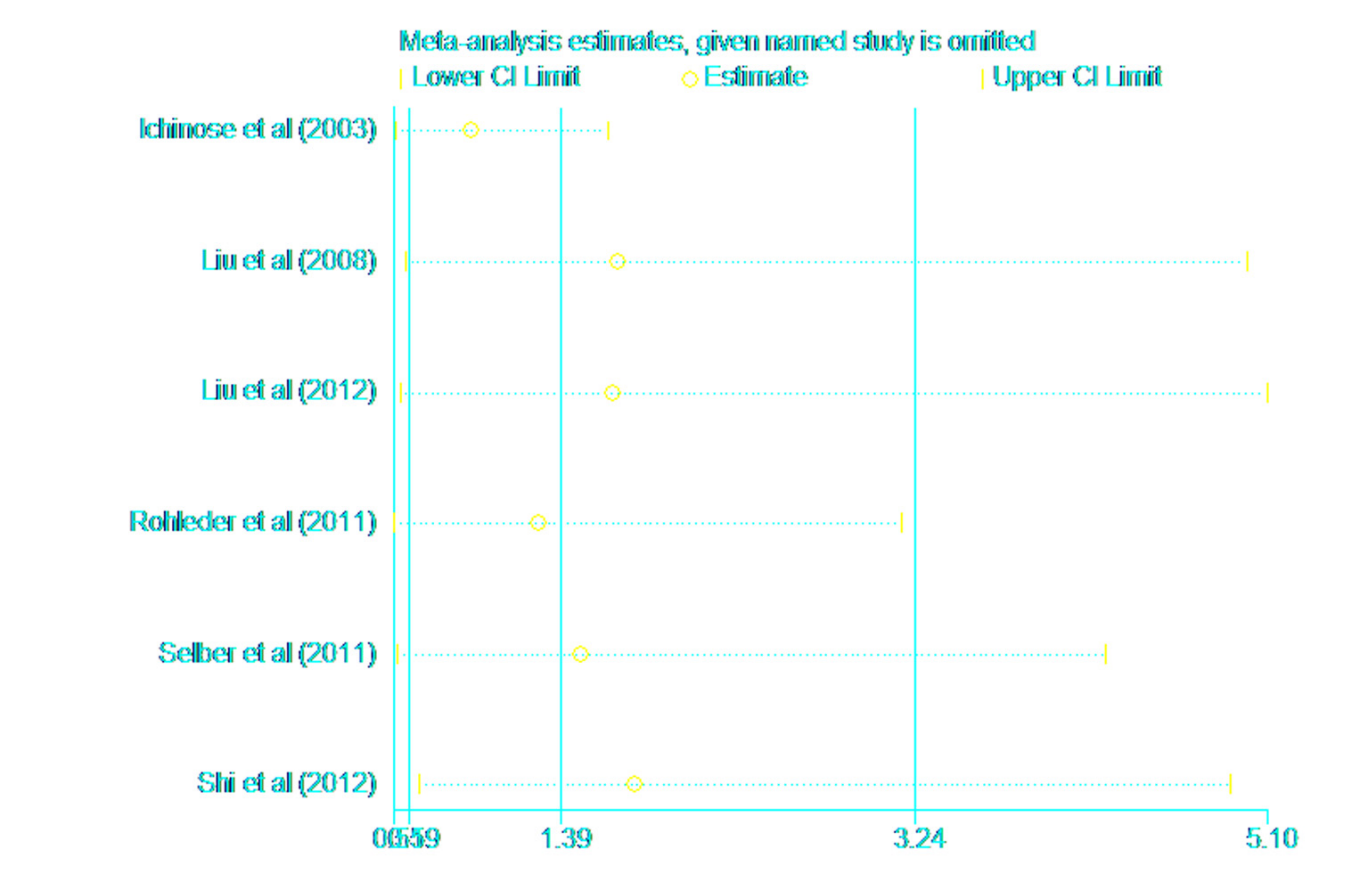
Results of the sensitivity analysis. The results of the sensitivity analysis showed that the study of Ichinose 2003 had a mild effect on the results.

The Begg and Egger rank correlation tests showed that there was no publication bias in this meta-analysis (P = 0.091, P = 0.133) (Fig 3). A sensitivity analysis was undertaken by removing a single study each time to reflect the influence to the pooled RR. The results of the sensitivity analysis showed that the study of Ichinose 2003 had a mild effect on the results. However, there was no individual study interfering with the overall pooled outcome apparently (Fig 4).

## Discussion

Since the initial description of the RFFF^2^, the superficial system was introduced as the primary venous drainage of this flap, although both systems were anastomosed.

Actually, in clinical practice, a sole cephalic vein anastomosis gains much preference because of the larger diameter when compared with the venae comitantes and the ease of performing anastomosis. Futran ND et al. [14] suggested that a single venous anastomosis employing the cephalic vein or a subcutaneous vein provided adequate drainage without additional morbidity, as well reduced operative time. Liu Y et al. [3] maintained that single superficial venous anastiomosis was anatomically or technically feasible to create and adequate to drain the radial forearm flap in their case series.

However, some clinicians occasionally obtain unfavorable results when employing a single cephalic vein anastomosis. Vaughn et al. [15] reported a 12.5 percent failure rate that was largely attributed to venous thrombosis of the superficial veins as the sole venous outflow of the RFFF. The cephalic vein has a thicker wall, the common weakness of the superficial veins, making it less prone to tears, rents and other mishandlings [3], especially which suffered venous damage from prior intravenous cannulation. Beckingham IJ et al. [16] reported a RFFF failure due to the cephalic vein occlusion secondary to previous intravenous cannulation, even though it appeared normal while be raised. All these unpredictable factors seem to make the single superficial vein drainage of RFFF unreliable.

Anatomical study showed that the superficial and deep venous system had no obvious communication in 60% cases [17]. Hence, some hypothesized that dual venous anastomoses would provide a fail-safe mechanism to afford a protection against venous insufficiency. Ichinose et al. [5] advocated a self-sustaining drainage system, which comprises the dual drainage veins of the superficial venous system and the deep system, and two independent recipient veins. Once one of parallel paths was occluded, the other might be influenced less. In fact, most authors [18] performed dual venous anastomosis from two different networks and believed that dual anastomosis on the same system (deep or superficial) would not improve significantly the venous drainage, compared with a single anastomosis.

The haemodynamic study has been demonstrated that the deep veins have twice the volume of drainage per unit time compared with the superficial vein [19]. Demirkan et al. [20] reported no venous compromise or partial/complete flap loss throughout the study of 94 consecutive RFFFs, using a single vena comitans anastomosis. It was proven to be as reliable as the cephalic vein or double vein anastomosis. Thus, we suppose that the high flap survival rate of the dual venous anastomoses might be largely due to the sufficiency of the deep vein system. The majority of current studies included veins selected from the same drainage system, both superficial or both deep veins; few reported the comparison between the two separate systems or the sufficiency of combination of superficial and deep systems. Hence, on the basis of present literature, subgroup analysis could not be performed.

The biggest concern from the supporters of the superficial veins is the inadequacy of the size of the deep veins. As a matter of fact, Shima et al. [21] have described the detailed vasculature of RFFF that the venae comitantes are comparable in size to the cephalic vein in the upper third of the forearm, even larger than the cephalic vein at the level of the antecubital fossa. Owing to the advance of the microsurgery technique, surgeons will spend just 20–30 minutes performing another anastomosis.

Various strategies for efficient drainage of the RFFF have been put forward, including a proximal dissection to the antercubital fossa near the confluence of the two venae comitantes, or the profundus cubitalis vein which may provide service of the two systems [22]. However, this communication between the deep and superficial systems has been found to be absent in 40% of cases [23]. This dissection would also create an unnecessarily lengthy pedicle prone to kinking. Thus, two anastomoses, one with the superficial and one with the deep veins, are recommended to decrease the risk of venous insufficiency.

Age and gender showed no significant difference in incidence of venous compromise. Age alone should not be considered a contraindication or an independent risk factor for free-tissue transfer [24]. Selber JC et al. [11] concluded that only cerebrovascular disease was significantly associated with an increased rate of venous complication in the univariate analysis. Other characteristics of the patients, such as tumor location showed no statistically difference in venous complication rate.

Angiography was not performed in the included studies. Because of sacrificing the radial artery, there is a potential risk contributing to the acute hand ischemia [25]. Fortunately, acute ischemic complications are exceedingly rare. Preoperative evaluation of the donor extremity including the Allen’s test, ultrasonography and angiography can evaluate arterial anatomy, patency, and communication between the radial and ulnar artery and avoid this potential vascular morbidity.

Microvascular anastomoses were performed with handmade interrupted sutures in all included cases. Some authors [26] have reported that there is no significant difference between handmade sutures and coupler anastomoses on the incidence of venous thrombosis and flap salvage rates following venous thrombosis proving the reliability of the coupler anastomoses.

Although the blood perfusion in the hand is not impaired in any clinically relevant way following flap harvest, as a main blood supply, the radial artery is sacrificed. Tendon exposure occurs occasionally. After the harvest of RFFF, a full-thickness skin graft will be performed, which creates a conspicuous scar, with mild, temporary weakness. As a whole, the long-term morbidity of this flap is low and easily managed.

The free medial sural artery perforator flap, thinned anterolateral thigh (ALT) flap and ulnar forearm free flap can serve as alternative flaps for small to medium-sized defect in the head and neck region after cancer ablation. For free medial sural artery perforator flap, donor-site morbidity, such as sensory disturbance, muscular weakness and scarring may occur [27]. The medial sural perforator artery and one venae comitant are anastomosed. Although two-team approach is performed, the position of the patient is also inconvenient for the surgeon to harvest the flap in head and neck reconstructive surgery. For thinned ALT flap, numbness of donor site and fatigue while climbing and walking upstairs may occur. The descending branch of the lateral circumflex femoral vessels is anastomosed. However, the perforator microdissection technique demands a steep learning curve. It may be time-consuming and may lose more than gain once damaging the perforator. Especially in the Western population, the ALT flap may be too bulky and in these cases the RFFF remains a better option [28]. For ulnar forearm free flap, donor site complications include subjective impression of reduced grip strength of the hand on the donor site, long-lasting reduced sensitivity or numbness in the ulnar site of the donor hands/forearms, even the symptom of ulnar nerve injury [29]. The ulnar artery and one venae comitant are anastomosed. Given to its thinness and pliability, the radial forearm flap is still the workhorse in head and neck reconstruction, especially in mobile tongue reconstruction.

**Fig 5:**
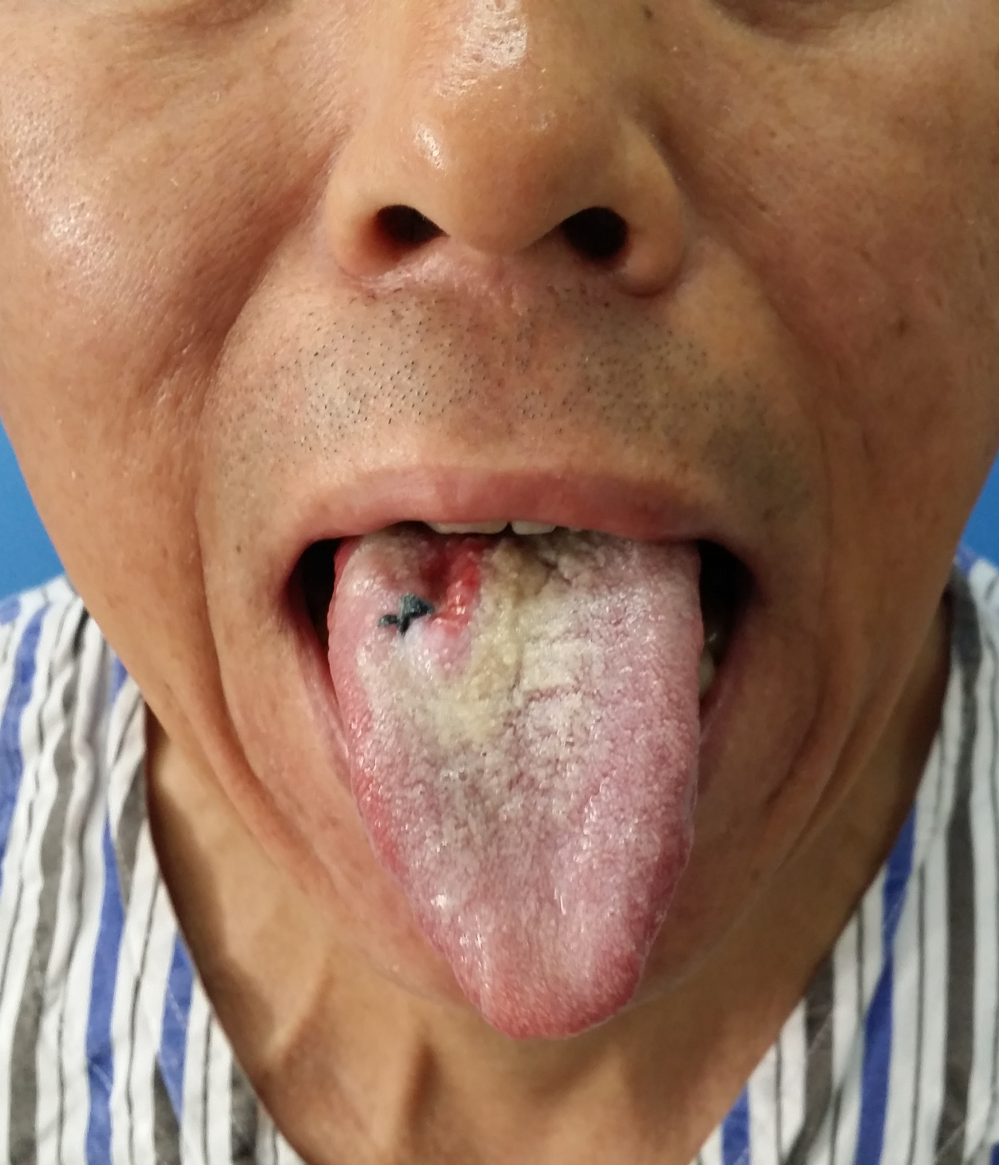
Preoperative view of the patient. The tumor involved the right base of the tongue till the midline.

**Fig 6:**
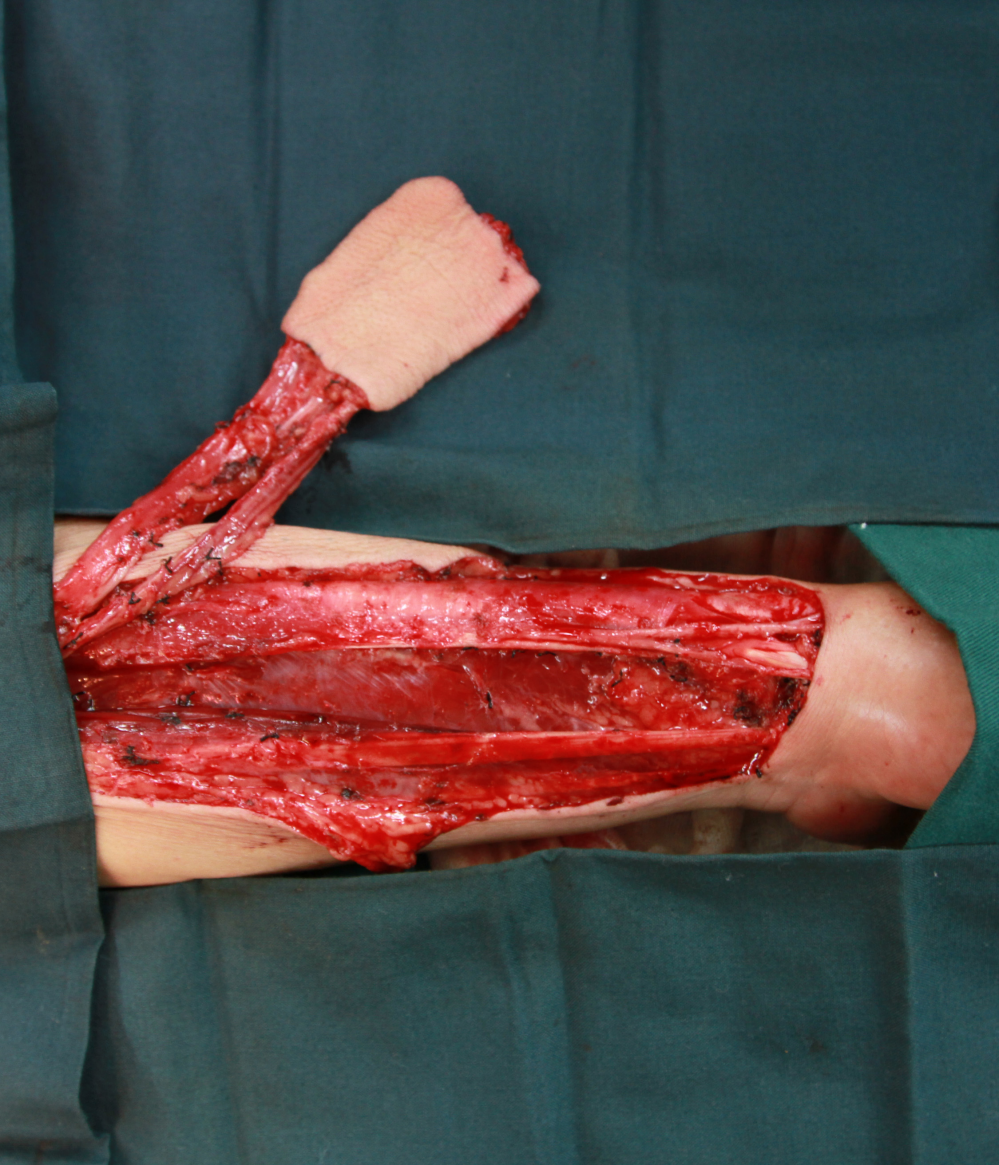
The intra-operative view of the flap harvesting. A RFFF was obtained from the patient’s left forearm.

**Fig 7:**
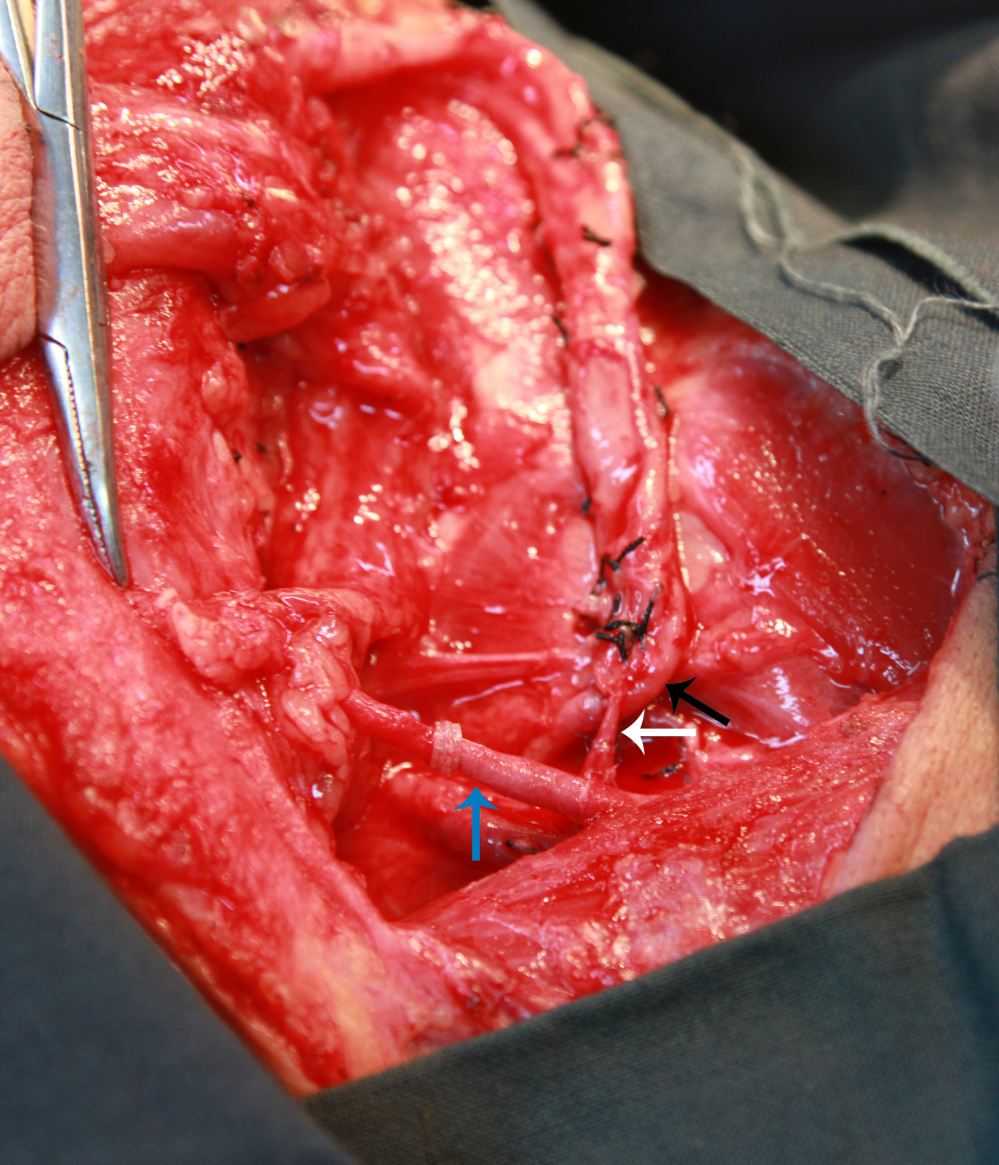
The strategy of venous anastomoses. Radial artery (black arrow), cephalic vein(blue arrow) and a venae comitant(white arrow) were anastomosed to the recipient vessels in the neck respectively.

**Fig 8:**
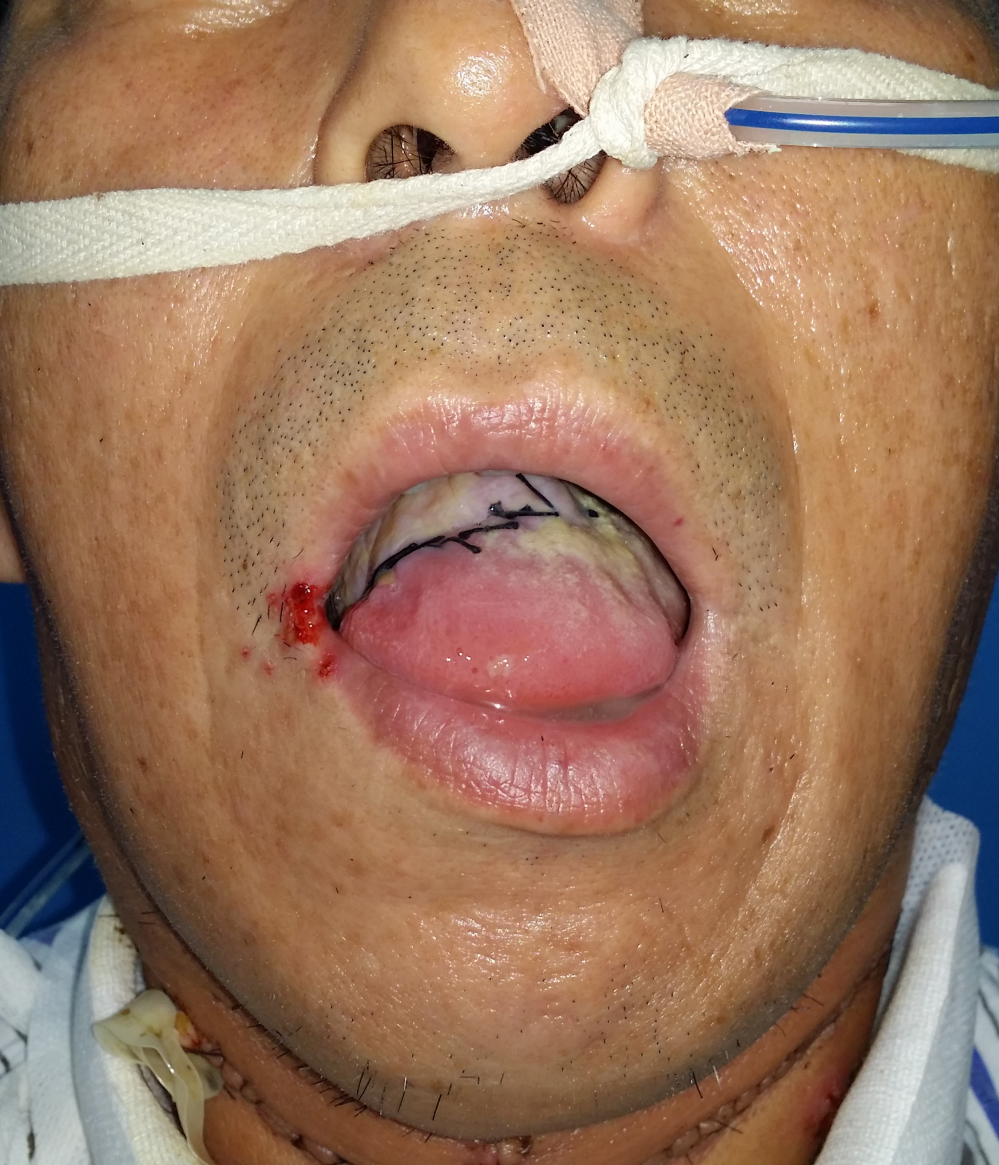
The postoperative view of the patient. The reconstruction of RFFF was uneventful 10 days after the operation.

In our experience, we prefer the dual venous anastomoses composed of cephalic vein and venae comitante. Thanks to the advance of our anastomosis technique, it only takes 15–25 minutes to accomplish a venous anastomosis by handmade suture and 5–7 minutes by coupler anastomosis. To be on the safe side, we perform two venous anastomoses from two system of RFFF. The success rate is over 97% in our center. 162 radial forearm flaps were performed at our department between 2005 and 2014. We will give a case presentation as following: a 49-year-old male presented to the Department of Oromaxillofacial-Head and Neck Surgery, School of Stomatology, China Medical University with right tongue mass, biopsy positive for squamous cell carcinoma (Fig 5). An extended tumor resection and bilateral selective neck dissection were performed. A radial forearm flap was obtained from his left forearm to reconstruct the tongue (Fig 6). Radial artery, cephalic vein and a venae comitant were chosen as the donor vessels and were anastomosed to the recipient vessels in the neck respectively (Fig 7). The postoperative course was uneventful, without serious complications (Fig 8).

Several limitations and sources of bias should be considered in this meta-analysis. First, only studies published in English and Chinese were searched in the process of study selection. The publication bias may exist, though there is no evidence of significant publication bias in this study, reflected by the test. Second, few study reported the comparison between the two separate systems or the sufficiency of combination of superficial and deep systems, the sample of this meta-analysis is not enough to perform a subgroup analysis. Although dual anastomosis consisting of the superficial and deep veins showed the tendency to have a lower incidence of venous compromise, the difference was not statistically significant. Thus, more original studies are needed. Furthermore, the included studies were most from Europe, North America and Asian. The absence of representative data from other part of world may exist, which made the results more prone to potential selection bias. Finally, the manipulation of anastomosis technique may probably be divergent between different clinical centers. This factor might potentially contribute to the better results of patients performed with dual system approach.

In conclusion, this meta-analysis indicated that performing dual venous anatomoses consisting of superficial and deep systems conferred a tendency of the reduction with regards to venous compromise. Our findings highlight the need for more studies to investigate the risk factors which lead to the venous thrombosis or flap loss. We suggest that the technique of dual anastomosis of venous drainage of the radial forearm flap should be performed, whenever the vasculature of the patient is suitable.

## Supporting Information

S1 Checklist PRISMA 2009 Checklist. (DOC)
